# Management and Visual Outcome in Patients of Lens-induced Glaucomas at a Tertiary Eye Care Hospital in South India

**DOI:** 10.5005/jp-journals-10008-1204

**Published:** 2016-08-05

**Authors:** M Sharanabasamma, K Vaibhav

**Affiliations:** 1 Assistant Professor, Department of Ophthalmology, Navodaya Medical College Hospital & Research Centre, Raichur, Karnataka, India; 2 Postgraduate and Junior Resident, Department of Ophthalmology, Navodaya Medical College Hospital & Research Centre, Raichur, Karnataka, India

**Keywords:** Lens-induced glaucoma, Phacolytic glaucoma, Phacomorphic glaucoma.

## Abstract

**Aims:** To outline the different characteristics of glaucomas and to determine the risk factors and their consequences on postoperative visual acuity, intraocular pressure (IOP), and inflammation, including corneal changes and optic disk changes.

**Settings and designs:** Longitudinal prospective study done over a period of 1.5 years in a medical college hospital.

**Materials and methods:** Fifty patients of lens-induced glaucoma (LIG) were included. At presentation, visual acuity, IOP, and inflammation, including corneal changes, were recorded. After medical line of treatment, postoperatively patients were followed up regularly at 2 and 7 weeks interval and the same parameters were evaluated including optic disk changes.

**Statistical analysis used:** Paired t-test, chi-square test wherever applicable with p-value < 0.05 as significant.

**Results:** The mean age of presentation was 60.68 years with female to male ratio of 1.7:1. The best corrected visual acuity(BCVA) of 6/18 or more was found in 54% cases, whereas visual acuity of less than 6/60 was seen in 26% of cases. Visual acuity of 6/12 or better was achieved in 72% (p < 0.01) of cases with symptoms less than 2 weeks and in 59.10% of cases with IOP of less than 35 mm Hg at presentation. The mean IOP in cases with duration of symptoms of 2 to 4 weeks was 40.33 ± 9.36 mm Hg. Optic disk of the affected eye suffered damage in 42% of cases and in 80% of cases with symptoms for more than 2 weeks.

**Conclusion:** Early diagnosis and treatment is beneficial in LIG cases.

**How to cite this article:** Sharanabasamma M, Vaibhav K. Management and Visual Outcome in Patients of Lens-induced Glaucomas at a Tertiary Eye Care Hospital in South India. J Curr Glaucoma Pract 2016;10(2):68-75.

## INTRODUCTION

In India, 62.6% blindness is due to cataract.^1^ Lens-induced glaucoma (LIG) is common in India.^2^ It is a common condition seen in patients with senile cataracts and is one of the commonest cause of secondary glaucoma, requiring an immediate attention and management to prevent blindness. These are heterogeneous group of disorders which develop through either open-angle or angle-closure mechanisms.^3^ Phacolytic glaucoma (PLG) and lens particle glaucoma are types of secondary open-angle glaucomas. The angle of anterior chamber is open with blockage of the trabecular meshwork by lens proteins. Phacomorphic glaucoma (PMG) and lens displacement glaucoma are types of secondary angle-closure glaucomas. Phacoanaphylactic uveitis, now termed as lens-induced uveitis, is not truly an anaphylactic reaction but is a granulomatous reaction that can cause open-angle or angle-closure glaucoma or combined open-angle and angle-closure glaucoma.^4^

There are various social, economic barriers for the people in our country for accessing excellent surgical services even these days. Whatever be the mode of surgical intervention, the prognosis for good postoperative visual recovery in these conditions remains guarded, unless diagnosed early and managed efficiently.^5^ Numerous studies have been done, which have determined and described the characteristics, risk factors, and causes for visual outcome and intraocular pressure (IOP) in LIG; also appropriate medical and surgical line of management have been formulated. Nevertheless, there are scanty reports and studies on the inflammation and its consequences in LIGs.

The present study has endeavored to determine the characteristics, risk factors, and their effects on postoperative visual outcome, inflammation, IOP including corneal changes and optic disk changes in LIGs.

## MATERIALS AND METHODS

Prospective type of study was done, which included 50 cases of different types of LIG, admitted in the ophthalmic wards of a medical college hospital from November 2011 to April 2013 after obtaining the Institutional Ethical Committee clearance.

### Inclusion Criteria

- All patients who were diagnosed having LIG
- Patients who signed written and informed consent for the surgery and also for the study were included.

### Exclusion Criteria

- Primary glaucoma
- Secondary glaucoma other than LIG
- Patients with poor general condition which made them unfit for surgery.

All patients diagnosed as LIG based on clinical symptoms and signs were included. Clinical features included pain, loss of vision, redness of the eye, headache, presence of an intumescent, mature or hyperma-ture cataract associated with raised IOP of more than 21 mm Hg.

A detailed clinical examination of both eyes included the status of the lens, depth of the anterior chamber by slit lamp biomicroscopy, and IOP recording by Perkins applanation tonometry, the values of which are comparable to Goldmann applanation tonometry. Based on the slit lamp examination, the type of LIG was determined. After patients signed the written and informed consent, the clinical features were noted in the study. At presentation, visual acuity, IOP, inflammation including corneal changes were recorded, which were again repeated after medical treatment. None of the cases had fundal glow at presentation.

The diagnosis of PMG was made in patients having redness of eye, with acute onset of pain and reduction of vision of certain duration. On clinical examination the eye showed circumcorneal congestion, corneal edema, shallow anterior chamber, dilated and fixed or sluggish pupil, intumescent cataract, and IOP more than 21 mm Hg.

Phacolytic glaucoma was diagnosed when patients presented with acute pain in the eye with long-standing poor vision. On examination, the eye showed marked diminution of vision, corneal edema, normal or deep anterior chamber containing floating lens particles, and/or with pseudohypopyon in severe cases, and hypermature morgagnian cataractous lens in few cases. Diffuse keratic precipitates and intense flare were seen.

The aim of treatment for these cases was to preserve useful vision, relief from pain, and reduction of the elevated ocular tension to almost normal levels, which was achieved by both medical and surgical methods. Analgesia was achieved by lowering down the IOP and by systemic administration of analgesics. Antiemetics were given in cases of severe vomiting.

### Preoperative Medical Line of Treatment

In all cases, preoperative medication to reduce IOP included either topical timolol 0.5% twice a day, oral acetazolamide 500 mg thrice a day, or intravenous man-nitol 20% alone or in combination, and also intravenous mannitol 20% was used before the surgery in refractory cases. Topical phenylephrine 10% was used just before surgery to achieve mydriasis so as to facilitate adequate capsulorhexis. Moxifloxacin eye drops were instilled in all the cases three times per day from the time of admission till the day of surgery as a prophylactic measure to make the conjunctival sac sterile. Systemic, oral ciprofloxacin (500 mg) 12 hourly was given as prophylaxis to prevent postoperative infection as intracameral antibiotics were not used.

### Surgical Line of Management

In all patients, cataract extraction with intraocular lens (IOL) implantation was offered under guarded prognosis. Irrespective of the level of fall of tension, all the patients were taken for surgery either on same day or on following day, depending upon patient’s condition. In all the patients, after peribulbar block, digital pressure was applied except in lens displacement cases, for nearly 8 to 10 minutes to achieve good hypotony. Planned manual small-incision cataract surgery (SICS) with IOL implantation was done in all but except in one case. In one case, after lens extraction IOL was not implanted, since the patient had posterior capsule tear, which needed an experienced vitreoretinal surgeon. Thorough anterior chamber wash with normal saline was given. At the end of the procedure, subconjunctival injection of steroid and antibiotic was given.

Postoperatively in the ward, all the patients received topical antibiotic - steroid combination - 1 drop hourly a day was given for mydriatic-cycloplegic effects, or twice a day if required. If severe uveal inflammation was present, then oral prednisolone 1 mg/kg body weight was given in a tapering dose over 3 weeks. If the tension was above 20 mm Hg, topical timolol 0.5% twice daily were instilled and in cases where tension was above 25 mm Hg, oral acetazolamide or IV mannitol 20% was given depending on the severity.

All the subjects were followed up regularly at day 1, day 14, and day 49. At every visit, patients were evaluated for visual acuity with Snellen’s chart, IOP by Perkins applanation tonometer, slit lamp examination of anterior segment, and posterior pole examination with direct ophthalmoscope and 90D lens.

The results were tabulated on Microsoft excel spreadsheet and the data was statistically analyzed using various tests, such as, paired t-test, chi-square test, and pooled chi-square test wherever applicable and a p-value of < 0.05 was considered significant.

## RESULTS

A total of 50 patients were included in the study. The age range was 46 to 75 years with a mean of 60.68 ± 6.65 years. The ratio between female and male was 1.7:1, with mean age of 60.41 ± 5.97 years in females and 61.17 ± 7.88 years among males. p-value is 0.237 which is not statistically significant. The most frequent age group among both males and females was 60 to 69 years. Phacomorphic glaucoma was the most common LIG with 43 cases (86%), followed by PLG with 7 cases (14%) (Table 1).

In this study, the mean duration of symptoms was 18.98 ± 16.22. The mean duration of symptoms in PMG cases was 18.97 ± 16.64 days and in PLG, it was 9 ± 14.52 day, which is insignificant statistically. Of the total 50 cases , 25 (50%) presented with symptoms of more than 2 weeks and 25 (50%) presented with duration less than 2 weeks. 22 (51.16%) of 43 PMG cases and 03 (42.86%) of 7 PLG cases presented with symptoms of more than 2 weeks.

No cases at presentation had visual acuity better than hand movements. At the last follow-up, 22 (44%) cases gained good visual acuity of 6/12 or better.

In this study, the total mean IOP at presentation was 38.88 ± 11.31 mm Hg (22-64 mm Hg); after medication, it was 18.76 ± 3.47 mm Hg, and at the last follow-up, it was 15.60 ± 2.10 mm Hg with the use of timolol 0.5% eye drops and tablet acetazolamide 250 mg. Paired t-test was used to compare the reductions of IOP from baseline at presentation to post medication, from baseline at presentation to last follow-up, and post medication to last follow-up. The respective mean were 20.12 ± 1.41 (t = 14.31, p < 0.0001), 23.32 ± 15.94 (t=14.62, p < 0.0001), and 2.78 ± 0.49 (t=5.65, p < 0.0001).

Inflammation at presentation was the most common and was mild to moderate in 32 cases (71.12%), while 12 cases had severe inflammation (26.66%). At discharge on day 3 after surgery, inflammation was reduced to mild in 22 cases (48.89%), to normal in 7 cases (15.56%), and only 4 cases had severe inflammation (08.89%). At the last follow-up on day 49, 41 cases (91.11%) were free of inflammation, none had severe inflammation, and rest had mild to moderate inflammation. The reduction of all grades of inflammation at presentation to normal on discharge was achieved in 7 (15.56%) cases, which was statistically significant [McNemar test, χ^2^=5.143, degrees of freedom (DF) = 1, p ≤ 0.05].

A majority of 48 patients (96%) underwent SICS with posterior chamber IOL implantation followed by 1 case with additional peripheral iridotomy done. Rest 1 case underwent only SICS without posterior chamber lens implantation.

In this study, a majority of 42 cases (93.34%) underwent extracapsular cataract extraction with posterior chamber IOL implantation.

At the last follow-up, 29 cases (58%) had normal fundus, 13 (26%) cases had 0.4 to 0.7 cup disk ratio (CDR), and 8 cases (16%) suffered severe glaucomatous disk damage (GDD) (0.8-0.9 CDR).

The best corrected visual acuity (BCVA) of 6/12 or better, at the last follow-up, was achieved in 10 (37.04%) cases of PMG and 8 (53.33%) cases of PLG (χ^2^ = 1.046, DF = 1, p ≥ 0.05). Poor visual outcome of less than 6/60 in both the subgroups were almost the same, with 8 (29.63%) cases in PMG and 4 (26.67%) cases in PLG (Table 2).

**Table 1:** **Table 1:** Distribution of cases according to duration of symptoms among LIG subgroups

		*Phacomorphic*				*Phacolytic*				*Total*			
*Duration (days)*		*No.*		*%*		*No.*		%		*No.*		%	
00-02		0		0		0		0		0		0	
03-07		12		27.91		1		14.29		13		26	
08-14		9		20.93		3		42.85		12		24	
15-30		13		30.23		1		14.29		14		28	
> 30		9		20.93		2		28.57		11		22	
Total		43		100.00		7		100.0		50		100	

**Table 2:** **Table 2:** Distribution of cases according to vision at presentation and BCVA at the last follow-up among LIG subgroups

*Phacomorphic glaucoma*												*Phacolytic glaucoma*												*Total of Vn at* * presentation*				*Total of* * BCVA*			
*BCVA*		*No.*		*%*		*Vn at* * presentation*		*No.*		*%*		*BCVA*		*No.*		*%*		*Vn at* * presentation*		*No.*		*%*		*No.*		*%*		*No.*		*%*	
6/6-6/12		18		41.86		HM		27		62.7		6/6-6/12		04		57.14		HM		5		71.42		32		64		22		44	
6/18-6/60		14		32.56		PL		14		32.5		6/18-6/60		01		14.29		PL		2		28.57		16		32		15		30	
< 6/60		11		25.58		PL		02		4.6		< 6/60		02		28.57		PL		0		0		2		4		13		26	
Total		43		100		Total		43		100		Total		07		100				07		100		50		100		50		100	

**Table 3:** **Table 3:** Distribution of cases according to BCVA at the last follow-up and duration of symptoms

*Duration of symptoms (in days)*																					
*BCVA*		*0-2*		*%*		*3-7*		*%*		*8-14*		*%*		*15-30*		*%*		*> 30*		*%*	
6/6-6/12		00		00		10		76.92		08		66.67		04		28.57		00		00	
6/18-6/60		00		00		03		23.08		04		33.33		06		42.86		02		18.2	
< 6/60		00		00		00		00.00		00		00		04		28.57		09		81.8	
Total		00		00		13		100.00		12		100.00		14		100.00		11		100	

The BCVA at the last follow-up of 6/12 or better was achieved in 16 (76.2%) of 21 cases with symptoms less than 2 weeks and in only 3 (12.5%) of 24 cases with symptoms of more than 2 weeks. The poor visual acuity at the last follow-up of less than 6/60 was found in 12 (50%) of 24 cases with symptoms more than 2 weeks and in 1 (04.16%) case with symptoms of less than 2 weeks. The duration of symptoms had linear relation with visual outcome. The more the duration of symptoms, the poorer the visual outcome (χ^2^/Yc = 15.205, p < 0.01) (Table 3).

The BCVA at the last follow-up of 6/12 or better was achieved in 9 (60%) cases that presented with IOP of less than 35 mm Hg, and in 10 (33.33%) cases with presentation IOP of more than 35 mm Hg. Poor vision of less than 6/60 was seen in 4 (26.67%) cases and 9 (30%) cases, with presentation IOP of less than 35 mm Hg and more than 35 mm Hg respectively (χ^2^ = 1.364, DF = 1, p > 0.05).

Good visual acuity at the last follow-up of 6/12 or better was achieved in 15 (46.88%) of 32 cases with mild to moderate inflammation at presentation and in 4 (30.77%) of 13 cases with severe to very severe inflammation. Poor visual acuity of less than 6/60 at the last follow-up was seen in 4 (12.5%) of 32 cases and 9 (69.23%) of 13 cases, with mild to moderate inflammation and severe to very severe inflammation respectively. The severity of inflammation at presentation was directly proportional to the poor visual outcome (χ^2^/Yc = 6.296, DF = 1, p < 0.05), which was statistically significant.

Seventeen (77.27%) of 22 cases whose fundus was normal and 2 (12.82%) of 18 cases with GDD achieved BCVA of 6/12 or better at the last follow-up. Whereas, 10 (55.5%) of 18 cases with GDD and none of normal fundus cases had visual acuity of less than 6/60. On applying Fisher’s exact test, the poor visual acuity of less than 6/60 in cases with GDD was found to be statistically significant (p = 0.000).

**Table 4:** **Table 4:** Distribution of cases according to IOP and duration of symptoms

				*Duration of symptoms (in days)*											
				*0-14 (n = 25)*				*15-30 (n = 14)*				*> 30 (n = 11)*			
*IOP*				*No.*		*%*		*No.*		*%*		*No.*		*%*	
At presentation		21-30		10		40		04		28.57		01		09.10	
		31-40		08		32		04		28.57		03		27.27	
		> 40		07		28		06		42.86		07		63.63	
After medication		00-20		23		92		11		78.57		09		81.8	
		21-30		02		08		03		21.43		02		18.2	
		> 31		00		00		00		00		00		00	
At the last		00-20		25		100		14		100		11		100	
follow-up		> 21		00		00		00		00		00		00	

At presentation, the mean IOP in cases with symptoms of less than 2 weeks was 38.76 ± 12.59 mm Hg. The mean IOP with symptoms for 2 to 4 weeks was marginally high with 40.33 ± 9.36 mm Hg and significantly high in cases with symptoms of more than 30 days with 44.67 ± 14.84 mmHg.

After medication, the mean IOP in cases with duration of less than 2 weeks was 22.48 ± 8.02 mm Hg. The mean duration of IOP with duration of 2 to 4 weeks was nearly equal to 22.88 ± 9.63 mm Hg and marginally high in cases with symptoms more than 30 days being 25.33 ± 13.24 mm Hg. At the last follow-up, the mean IOP with symptoms of less than 2 weeks was 14.09 ± 1.73 mm Hg and was nearly equal in cases with symptoms for 2 to 4 weeks and more than 30 days being 18.44 ± 6.98 and 18.67 ± 12.43 mm Hg respectively.

The mean IOP at presentation in PMG was 40.67 ± 12.96, and 39.87 ± 10.62 mm Hg in PLG. After medication, the mean IOP was 23.37 ± 8.78 and 20.67 ± 6.91 mm Hg and at the last follow-up it was 16.67 ± 5.71 and 16.93 ± 8.07 mm Hg respectively. These differences are neither clinically nor statistically significant (Table 4).

At presentation, the PMG commonly presented with mild inflammation in 12 cases (44.46%) compared to PLG in 3 (20.00%) cases, which commonly presented with severe inflammation in 6 cases (40.00%) as against 5 cases (18.51%) in PMG. The inflammation reduced to normal with preoperative medication in 3 cases (11.11%) of PMG and 4 cases (26.67%) of PLG respectively. At the end of 6th week follow-up, the inflammation subsided almost to normal in 25 cases (92.50%) and 13 cases (86.66%) of PMG and PLG respectively. None of the total cases had severe inflammation at the end of last follow-up. The difference in inflammation among subgroups at presentation (χ^2^/Yc = 2.491, DF = 1, p > 0.05) and after medication (χ^2^/Yc = 0.8, DF = 1, p > 0.05) was statistically not significant (Table 5).

The inflammation was mild to moderate in 17 (80.95%) of 21 cases with duration of symptoms less than 2 weeks and in 15 (71.42%) of 24 cases with duration of symptoms more than 2 weeks, whereas severe to very severe inflammation was found in 4 (19.04%) of 21 cases and 9 (37.50%) of 24 cases respectively. There was statistically significant correlation between severity of inflammation and duration of symptoms (χ^2^=8.008, DF=1, p < 0.05) (Table 6).

Inflammation at presentation was mild to moderate in 14 (93.33%) cases with IOP less than 35 mm Hg and in 18 (60%) cases with IOP more than 35 mm Hg. Severe to very severe inflammation was seen in 1 (6.67%) case and 12 (40%) cases with IOP less than 35 and more than 35 mm Hg respectively (χ^2^=9.504, DF=1, p < 0.05) (Table 7).

**Graph 1:**
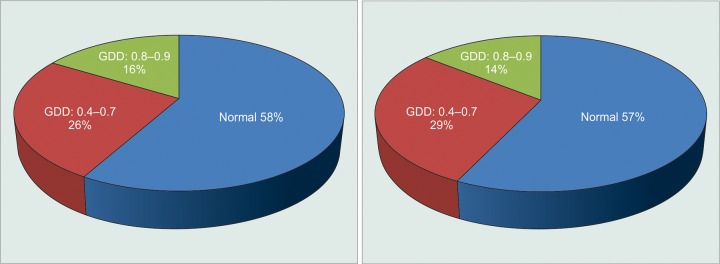
Distribution of cases according to optic disk changes among LIG subgroups

The optic disk changes at the end of last follow-up were normal in 11 cases (40.75%) and 9 cases (60%) in PMG and PLG respectively (Graph 1). Mild to moderate GDD was seen in 7 cases (25.93%) of PMG and in only 4 cases (26.66%) of PLG, whereas severe damage was seen in 6 cases (22.22%) and 1 case (06.67%) respectively. The difference in the optic disk appearance among subgroups was statistically not significant (χ^2^ = 1.434, DF = 1, p > 0.05).

**Table 5:** **Table 5:** Distribution of cases according to inflammation among LIG subgroups

				*Lens-induced glaucoma*							
				*Phacomorphic (n = 43)*				*Phacolytic (n = 7)*			
*Inflammation*				*No.*		*%*		*No.*		*%*	
At presentation		Mild		18		41.86		00		00.00	
		Moderate		14		32.56		04		57.14	
		Severe		11		25.58		02		28.57	
		Very severe		00		00.00		01		14.29	
		Normal		08		18.6		00		00	
On discharge		Mild		17		39.5		03		42.86	
		Moderate		12		27.9		03		42.86	
		Severe		06		14.0		01		14.28	
		Very severe		00		00.0		00		00.00	
		Normal		43		100		06		85.72	
Last follow-up		Mild		00		00		01		14.28	
		Moderate		00		00		00		00	
		Severe		00		00		00		00	
		Very severe		00		00		00		00	

**Table 6:** **Table 6:** Distribution of cases according to inflammation at presentation and duration of symptoms

		*Duration of symptoms (in days)*																			
		*0-2 (n = 0)*				*3-7 (n = 13)*				*8-14 (n = 12)*				*15-30 (n = 14)*				*> 30 (n = 11)*			
*Inflammation at* *presentation*		*No.*		*%*		*No.*		*%*		*No.*		*%*		*No.*		*%*		*No.*		*%*	
Mild		00		00		10		77		04		33.33		04		28.6		00		00.00	
Moderate		00		00		02		15		06		50.00		05		35.7		05		45.45	
Severe		00		00		01		08		02		16.67		05		35.7		05		45.45	
Very severe		00		00		00		00		00		00		00		00		01		09.10	

**Table 7:** **Table 7:** Distribution of cases according to inflammation at presentation and IOP

		*IOP*							
		*< 35 mm Hg*				*>35 mm Hg*			
*Inflammation at* *presentation*		*No.*		*%*		*No.*		*%*	
Mild		12		54.54		06		21.43	
Moderate		08		36.36		10		35.71	
Severe		02		09.10		11		39.29	
Very severe		00		00		01		03.57	
Total		22		100		28		100	

Fundus at the last follow-up was found to be normal in 17 cases (80.95%) presented before 2 weeks and in 5 (20.83%) of 24 cases presented later than 2 weeks. Mild to moderate disk damage was found in 8 (44.45%) cases of those presented between 2 and 4 weeks and severe damage was found in 5 cases (83.33%) of those presented more than 30 days. There was statistically significant correlation between severity of damage to the disk and duration of the symptoms (χ^2^=16.200, DF = 1, p < 0.01) (Table 8).

**Graph 2:**
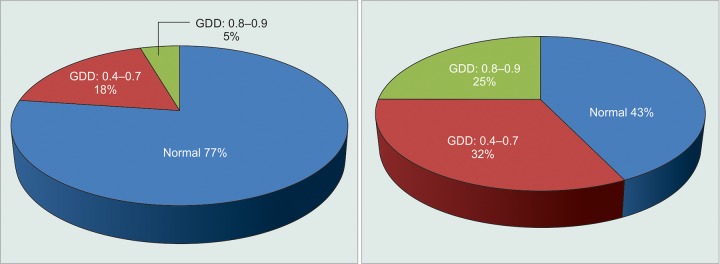
Optic disk changes by IOP

Fundus changes at the last follow-up were normal in 9 cases (60%) of those presented with IOP less than 35 mm Hg, and in 13 cases (43.33%) of those presented with IOP more than 35 mm Hg. There was severe GDD in 1 case (06.67%) and 6 cases (20.00%) of those presented with IOP less than 35 mm Hg and more than 35 mm Hg respectively (Graph 2). The correlation between severity of disk damage and height of IOP was statistically not significant (χ^2^=1.11, DF = 1, p > 0.05).

**Table 8:** **Table 8:** Distribution of cases according to optic disk changes and duration of symptoms

		*Duration of symptoms (in days)*											
		*0-14*				*15-30*				*> 30*			
*Optic disk*		*No.*		*%*		*No.*		*%*		*No.*		*%*	
Normal		24		96		05		35.71		00		00.00	
GDD: 0.4-0.7		01		04		06		42.86		06		54.55	
GDD: 0.8-0.9		00		00		03		21.43		05		45.45	
Total		25		100		14		100		11		100	

## DISCUSSION

Lens-induced glaucomas occur commonly in India. Though these are clinically distinct entities, they have certain common factors in that they are resultant of lens and damage the optic nerve because of high IOP with cataract surgery as definitive treatment in these cases, and finally they uniformly share a guarded prognosis.^6^

This longitudinal study was undertaken to study the management of LIG and to study the visual outcome after planned manual SICS.

In this study, the age range was 46 to 75 years with a mean age of 60.68 ± 6.65 years. Highest number of cases occurred in the age group 60 to 69 years (60%). In Lahan study, it has been found that the occurrence of LIG is in the age range of 40 to 80 years and highest in the 60 to 69 years (43.1%) age group, indicating that the LIGs are a condition of old age.^5^

Females had an increased risk of LIG over males with a ratio of 1.7:1 in this study, which is comparable to that of a study conducted in Madurai with female pre-ponderence (p = 0.05).^6^ The ratio between females and males in Lahan study was 1.7:1. It is possible that these entities are more common in females because of socioeconomic repression, and also because of the fact that cataract is more prevalent among females than males. This finding was consistent with data from the Punjab study in India and from the MATLAB study in Bangladesh.^5^

Lens-induced glaucomas are either angle-closure or open-angle type, resulting due to some disorder of crystalline lens.

Phacomorphic glaucoma is defined as a secondary angle-closure glaucoma due to lens intumescence. An acute rise of IOP can hamper the optic nerve function and may lead to irreversible visual loss if not treated on time.

Phacolytic glaucoma is the sudden onset of open-angle glaucoma caused by a leaking mature or hypermature cataract.

The observations made in this study conclude that PMG (86%) was the most frequent type of LIG followed by PLG (14%). Similar occurrence was noted in Madurai study (52.68%)^6^ and Lahan study (72%).^5^ Occurrences of various LIGs in the above studies show variations. Almost always, PMG is the most common type of LIG among several studies, even the present one, which is peculiar to the developing countries. In this study, none of PMG occurred below 50 years of age, showing that PMG is a disease of old age with preponderance in 60 to 69 years age group. This may be due to the unawareness and lack of medical services in developing countries. In contrast, PLG represents lens-induced acute secondary open-angle glaucoma associated with quicker onset of pain, redness, and watering in the eye with an acute elevation of IOP, making the patient to seek medical advice earlier than PMG.

In this study, none of the cases had vision better than hand movement at presentation. In the present study, BCVA of 6/18 or better is slightly higher (54%) when compared with Lahan study series (31.40%), and much higher when compared with Pravara Rural Hospital, Loni study (16%). Thus, in this study, higher percentage of cases has achieved good visual recovery and lower percentage of cases have poor visual outcome when compared to Lahan study series^5^ at Pravara Rural Hospital, Loni.^7^

The BCVA in this study of 6/12 or better was low (44%) and poor vision of less than 6/60 higher (26%) compared to Madurai study, with 59.13 and 11.82% respectively.^6^ The BCVA of 6/12 or better was taken as good visual acuity and less than 6/60 as poor visual outcome.

In this study, good visual acuity achieved by cases with PLG (57.14%) was more than PMG (41.86%) and this difference was clinically significant but statistically not significant (p > 0.05). Poor outcome of less than 6/60 showed no significant difference between PLG (28.57%) and PMG (25.58%). There was no statistically significant difference noted among the two groups on final postoperative visual recovery in Madurai study (p = 0.68).^6^

Good visual acuity achieved in cases presented within 2 weeks (72%) was more than the cases presented beyond 2 weeks (16%), whereas poor visual acuity of less than 6/ 60 was more in cases presented beyond 2 weeks (52%). Our study has a linear relationship between the symptom duration and BCVA at final follow-up. As there was delay in the initial presentation, the visual outcome became more guarded, which was clinically and statistically significant (p < 0.01).

The Lahan study of 1998 found that the duration of pain and high level of IOP at presentation in PMG was associated with poor visual outcome at discharge, while in PLG no such association were made out.^5^

In our setup, the patients presented late, probably because of poverty, ignorance, lack of awareness, facilities for treatment, quackery at peripheries, and lack of prompt referral and helplessness of patients would have led to increase morbidity.

In this study, the influence of inflammation on final visual outcome was analyzed. Good visual acuity achieved in cases with mild to moderate (52.78%) inflammation was higher than cases with severe to very severe inflammation (21.43%). Poor vision was found to be higher in cases with severe inflammation (71.43%) than with mild to moderate inflammation (8.33%). The final visual acuity was affected by the severity of inflammation which was significant both clinically and statistically (p < 0.05).

Madurai study had found no statistically significant association between the level of preoperative IOP and final visual acuity.^6^

The IOP reduction from baseline at presentation to post medical line of treatment and to last follow-up and from medical line of treatment to last follow-up were found to be clinically and statistically significant. It is found that the reduction in mean IOP is greater after medication for glaucoma. Nevertheless, surgical removal of lens is the definite treatment for LIG and response to medications is very good in LIG. The IOP at the last follow-up was reduced to normal limits (16.44 ± 6.54 mm Hg). This indicates that in LIGs, IOP should be reduced by medical line of management prior to surgery near-normal to normal to achieve stable IOP postoperatively without any further antiglaucoma medications. Intraocular pressure tends to be higher if there is a delay in presentation beyond 30 days (44.67 ± 14.84 mm Hg) than the duration of presentation of less than 2 weeks (38.76 ± 12.59 mm Hg). The mean preoperative IOP was 46.2 mm Hg in study by Kothari et al^7^ similar to that of our study. Though the mean IOP at the last follow-up was normal (16.44 ± 6.54 mm Hg), cases with delay in presentation between 2 and 4 weeks and more than 30 days tend to be on higher end of normal (18.44 ± 6.98 and 18.67 ± 12.48 mm Hg). So a delay of more than 2 weeks in presentation would result in higher IOP, notably if the delay is beyond 30 days, which is clinically significant.

Lahan study has achieved slightly better IOP control than our study, especially after antiglaucoma medication.^5^ Madurai study results were similar to our study regarding IOP presentation and at follow-up.^6^ The 1991 Delhi study on PMG was not able to control IOP in 37.5% eyes.^8^ The 1990 Vellore study on PLG has found no significant correlation between duration of symptoms and the presenting IOP, as in the present study. There was a weak positive correlation found between the duration of symptoms and the best-controlled preoperative IOP, similar to the present study.^9^

Optic disk at the last follow-up in the affected eyes was normal in majority (58%) of cases. Glaucomatous disk damage was found in 42% cases. Though clinically significant disk damage was found more in PMG (41.86%) than in PLG (42.85%), the difference was statistically not significant (p > 0.05). There was significant damage in cases presented beyond 2 weeks (64.28%) and especially beyond 30 days (100%) than in cases presented before 2 weeks (4%). Fundus was normal in majority (96%) of cases presented before 2 weeks. The severity of GDD was directly proportional to the duration of symptoms, which was clinically and statistically significant (p < 0.01).

The BCVA at the last follow-up correlated with fundus changes and has shown that the good visual acuity achieved in normal fundus (68.97%) was significantly high than in cases with GDD (915.4%). In addition, poor visual acuity in cases with GDD was definitely high (61.90%) as none of the cases with normal fundus had poor visual acuity which were clinically and statistically significant (p = 0.0001).

In Madurai study, 5 patients with PMGs and 4 patients with PLGs had compromised optic nerves due to the glaucomatous process itself, showing close association with our study inference.^6^ In Lahan study series, the percentage of optic atrophy cases (34.0%) is comparatively high from our study (15.56%).^5^

## CONCLUSION

The LIGs are a condition of old age with increased risk in females. The results have shown that good visual acuity can be achieved in LIG presenting within 2 weeks, with IOP of less than 35 mm Hg and with meticulous control of IOP and inflammation with medications preopera-tively. Planned manual small-incision cataract extraction with IOL implantation, minimal tissue handling, a good follow-up with efficient management of attendant complications, and inflammation are the key factors in the management.

Delay in presentation of more than 2 weeks and IOP of more than 35 mm Hg would result in severe inflammation, affecting the optic nerve, which would ultimately imperil vision in these potentially blinding LIGs. Emphasis should be made on creating awareness about cataract, its implications among the rural community, ophthalmic assistants, and peripheral health workers. This study has highlighted the characteristics, risk factors, and their consequences in LIGs, and also the importance of early diagnosis and efficient medical management to control IOP and inflammation, with meticulous surgery and IOL implantation. Also proficient postoperative management and follow-up would probably achieve excellent visual prognosis.
